# Distinct Gene Set Enrichment Profiles in Eosinophilic and Non-Eosinophilic Chronic Rhinosinusitis with Nasal Polyps by Bulk RNA Barcoding and Sequencing

**DOI:** 10.3390/ijms23105653

**Published:** 2022-05-18

**Authors:** Takashi Ishino, Sachio Takeno, Kota Takemoto, Kensuke Yamato, Takashi Oda, Manabu Nishida, Yuichiro Horibe, Nobuyuki Chikuie, Takashi Kono, Takayuki Taruya, Takao Hamamoto, Tsutomu Ueda

**Affiliations:** Department of Otorhinolaryngology, Head and Neck Surgery, Graduate School of Biomedical Sciences, Hiroshima University, Kasumi 1-2-3, Hiroshima 734-8551, Japan; tishino@hiroshima-u.ac.jp (T.I.); kota61@hiroshima-u.ac.jp (K.T.); kkyy7410@hiroshima-u.ac.jp (K.Y.); odataka@hiroshima-u.ac.jp (T.O.); nm1027@hiroshima-u.ac.jp (M.N.); horibey@hiroshima-u.ac.jp (Y.H.); housejak@hiroshima-u.ac.jp (N.C.); tkono@hiroshima-u.ac.jp (T.K.); ttaruya@hiroshima-u.ac.jp (T.T.); takao0320@hiroshima-u.ac.jp (T.H.); uedatsu@hiroshima-u.ac.jp (T.U.)

**Keywords:** paranasal sinuses, chronic rhinosinusitis (CRS), BRB-seq, CRS endotypes, nasal polyps, eosinophils, differentially expressed genes (DEGs) analysis, database for annotation, visualization and integrated discovery (DAVID), pathway analysis, type 2 inflammation

## Abstract

Chronic rhinosinusitis with nasal polyps (CRSwNP) is a chronic inflammatory disease with a high symptom burden, including nasal congestion and smell disorders. This study performed a detailed transcriptomic analysis in CRSwNP classified as eosinophilic CRS (ECRS), nonECRS according to the Japanese Epidemiological Survey of Refractory Eosinophilic Chronic Rhinosinusitis (JESREC) criteria, and a group of ECRS with comorbid aspirin intolerant asthma (Asp). Gene expression profiles of nasal polyps and the uncinate process in CRSwNP patients and normal subjects (controls) were generated by bulk RNA barcoding and sequencing (BRB-seq). A differentially expressed genes (DEGs) analysis was performed using DESeq2 software in iDEP to clarify any relationship between gene expression and disease backgrounds. A total of 3004 genes were identified by DEGs analysis to be associated with ECRS vs control, nonECRS vs control, and Asp vs control. A pathway analysis showed distinct profiles between the groups. A Kyoto Encyclopedia of Genes and Genomes (KEGG) pathway analysis using the Database for Annotation, Visualization, and Integrated Discovery (DAVID) showed distinct phenotype-specific pathways of expressed genes. In the specific pathway of “cytokine–cytokine receptor interaction”, the differentially expressed genes were widely distributed. This study indicates that transcriptome analysis using BRB-seq may be a valuable tool to explore the pathogenesis of type 2 inflammation in CRSwNP.

## 1. Introduction

Chronic rhinosinusitis (CRS) is a chronic inflammatory upper airway disease with a high symptom burden, including nasal congestion, smell disorders, nasal discharge, and facial pain/pressure [1,2]. Based on the presence or absence of nasal polyps, CRS can be classified as without nasal polyps (CRSsNP) or with nasal polyps (CRSwNP), which have distinct inflammatory profiles and pathogenesis. CRS can be subclassified into further categories by its underlying cellular and molecular pathophysiologic mechanisms [3], and CRSwNP can also be subclassified into ECRS and nonECRS in terms of the degree of eosinophilic infiltration of sinonasal tissue [4]. Compared with nonECRS, which is supposed to be characterized by Th1-dominant inflammation, ECRS is a phenotype of CRSwNP characterized by Th2-dominant inflammation, which is difficult-to-treat and leads to poorer surgical outcomes [4,5]. Additionally, ECRS patients with comorbid aspirin intolerant asthma (AIA) are thought to have more difficult-to-treat CRS due to more severe type 2 inflammation [4,6]. Therefore, the type of inflammation in CRSwNP is thought to be a key factor in classifying CRSwNP as a difficult-to-treat CRS. Currently, JESREC criteria are used to classify ECRS into mild, moderate, and severe ECRS [4]. From a transcriptome analysis of nasal polyps, it was certified that nasal polyps can be segregated into two major endotypes showing a type 2 or non-type 2 endotype [7]. However, a previous study using a transcriptome analysis with three ECRS, three nonECRS, and three control subjects showed no distinct differentiation of expressed mRNAs between ECRS and nonECRS [8]. Given the discrepancy between the inflammatory differences of CRSwNP and differentiation of expressed mRNAs in transcriptome analysis, we presume that the severity of eosinophilic infiltration does not enhance the severity of type 2 inflammation or pathological endotype. This study performed a detailed transcriptomic analysis among ECRS and nonECRS based on the JESREC criteria and a group of ECRS with comorbid AIA (Asp) to reveal whether the diagnosis of ECRS and/or Asp correlates with the severity of type 2 inflammation or pathological endotype. Because RNA-seq, a method for transcriptomic analysis, is still too laborious and expensive, we used bulk RNA barcoding and sequencing (BRB-seq), which is a comparatively novel method for RNA sequencing and uses early multiplexing to produce 3′ cDNA libraries for dozens of samples [9]. BRB-seq has a comparable performance to the standard TruSeq approach while showing greater tolerance for lower RNA quality and being up to 25 times cheaper [9]. In this study, we applied unsupervised clustering methods from comprehensive RNA gene expression data using BRB-seq and analyzed the difference between these three groups of CRSwNP to gain insights into the molecular profiles and endotype categorization of CRSwNP.

## 2. Results

### 2.1. Gene Expression Profiles of NPs between ECRS, NonECRS, and Asp

#### PCA, Heatmap of the Correlation Matrix, and Hierarchical Cluster Analysis

**Figure 1:**
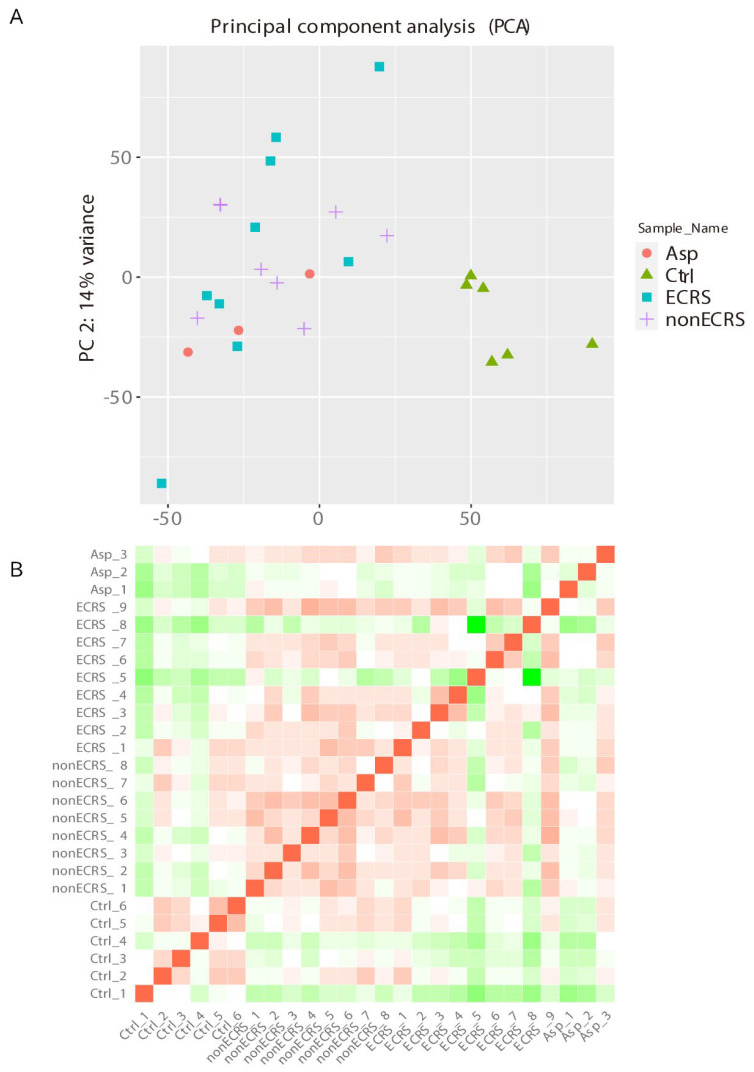
PCA and heatmap of the correlation matrix performed among all groups. The control group was clearly segregated from the other groups. ECRS, nonECRS, and Asp were not segregated from each other (**A**). The heatmap of the correlation matrix also showed the difficulty of the segregation among the three CRSwNP groups (**B**).

PCA was performed among all groups and revealed that the control group was clearly segregated from the other groups, but ECRS, nonECRS, and Asp were not segregated from each other (Figure 1A). A heatmap of the correlation matrix also clarified the difficulty of the segregation among the three CRSwNP groups (Figure 1B). 

**Figure 2:**
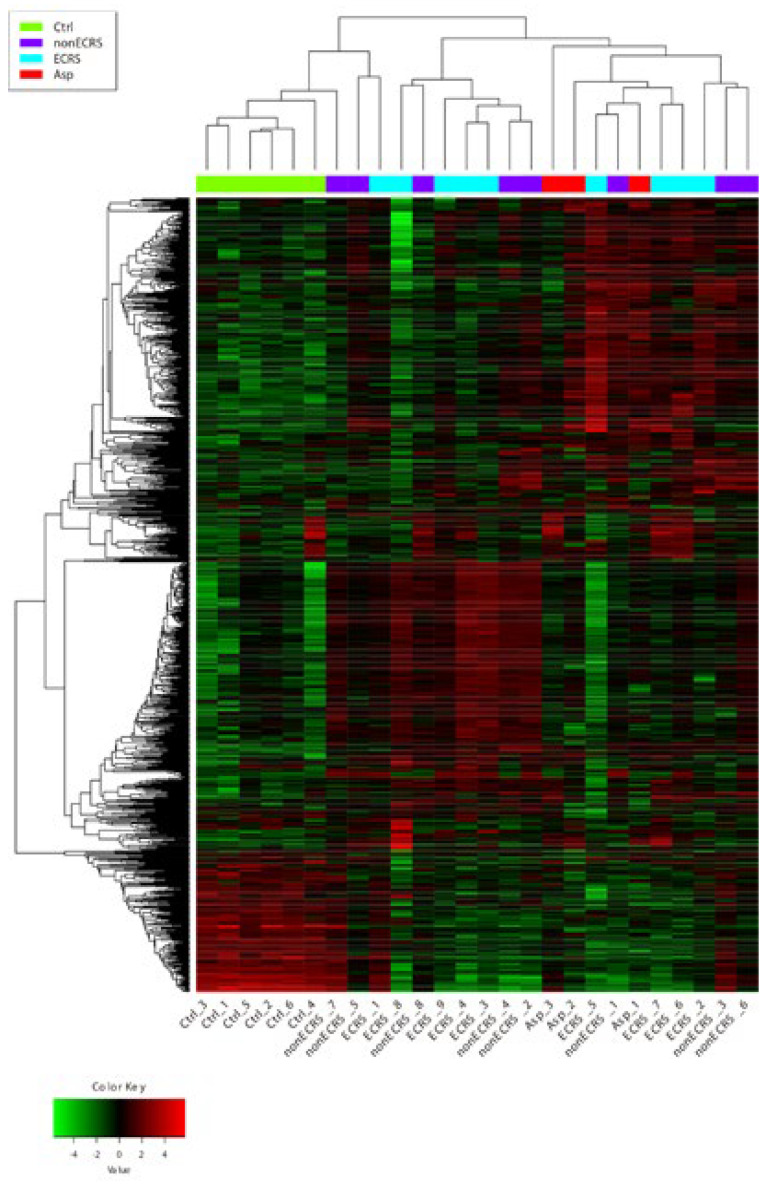
Hierarchical cluster analysis with cutoff Z score of 4. No distinct segregation among the three CRSwNP groups was seen and some ECRS (ECRS1) and nonECRS (nonECRS5 and 7) patients were in a similar cluster to the control group.

**Figure 3:**
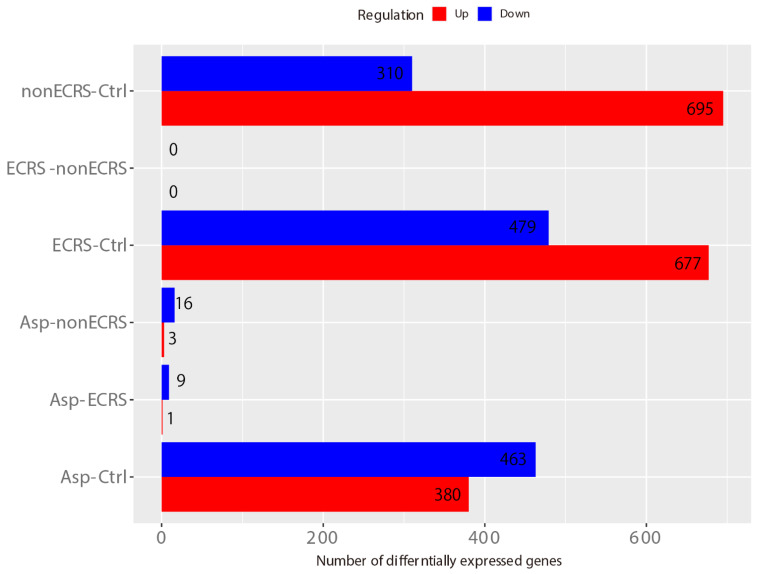
DEGs in ECRS vs. control (ECRS-Ctrl), 677 upregulated and 479 downregulated; nonECRS vs Ctrl (nonECRS-Ctrl), 695 and 310; Asp vs Ctrl (Asp-Ctrl), 380 and 463; Asp vs nonECRS (Asp-nonECRS), 3 and 16; and Asp vs ECRS (Asp-ECRS), 1 and 9. No DEGs were identified in ECRS vs nonECRS (ECRS-nonECRS).

Hierarchical cluster analysis with a cutoff Z score of 4 revealed no distinct segregation among the three CRSwNP groups, and some ECRS (ECRS1) and nonECRS (nonECRS5 and 7) patients were clustered with the control groups (Figure 2). DEGs were identified as both upregulated and downregulated genes in ECRS vs control (ECRS-Ctrl) of 677 upregulated and 479 downregulated, nonECRS vs control (nonECRS-Ctrl) of 695 and 310, Asp vs control (Asp-Ctrl) of 380 and 463, Asp vs nonECRS (Asp-nonECRS) of 3 and 16, and Asp vs ECRS (Asp-ECRS) of 1 and 9. However, no DEGs were identified in ECRS vs nonECRS (ECRS-nonECRS) (Figure 3).

**Figure 4:**
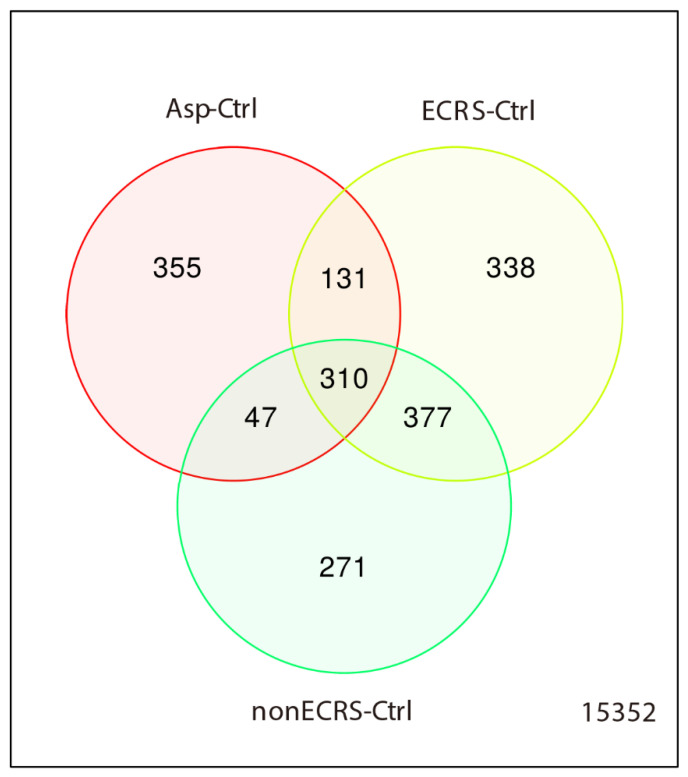
Venn diagram of all CRSwNP groups vs control. Specific DEGs were identified in each CRSwNP group as ECRS-Ctrl of 338, nonECRS-Ctrl of 271, and Asp-Ctrl of 355.

However, a Venn diagram of all CRSwNP groups vs control showed the existence of specific DEGs in each CRSwNP group (Figure 4). 

**Figure 5:**
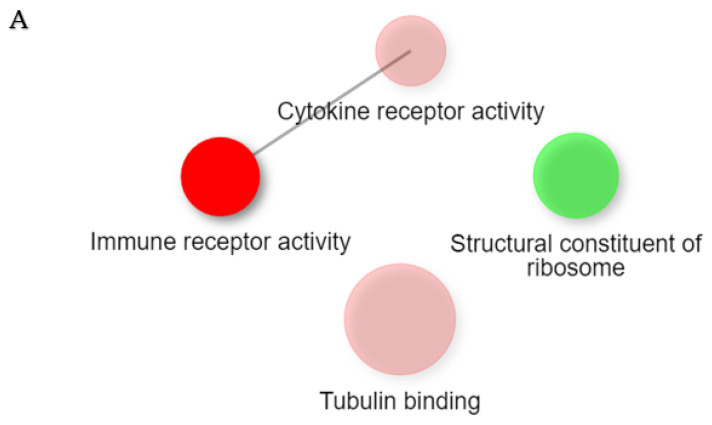

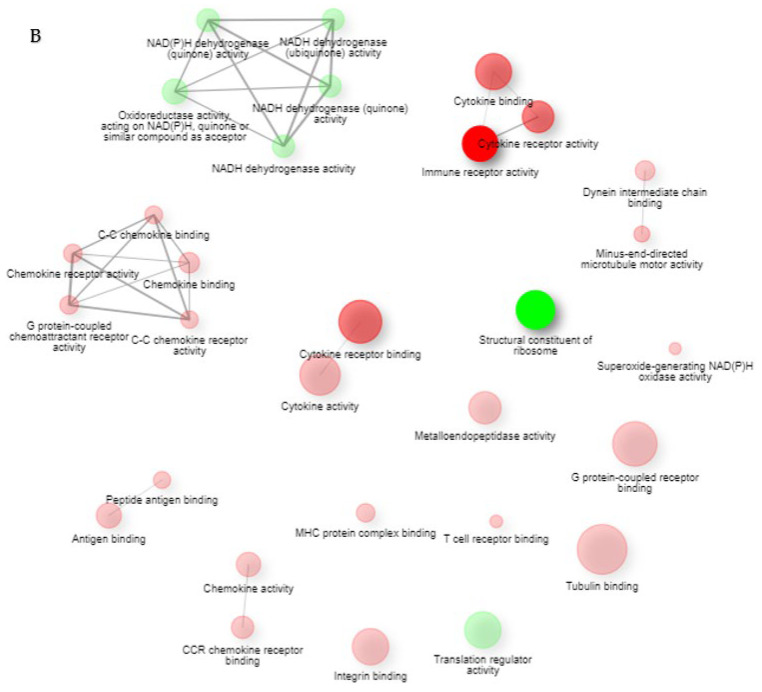

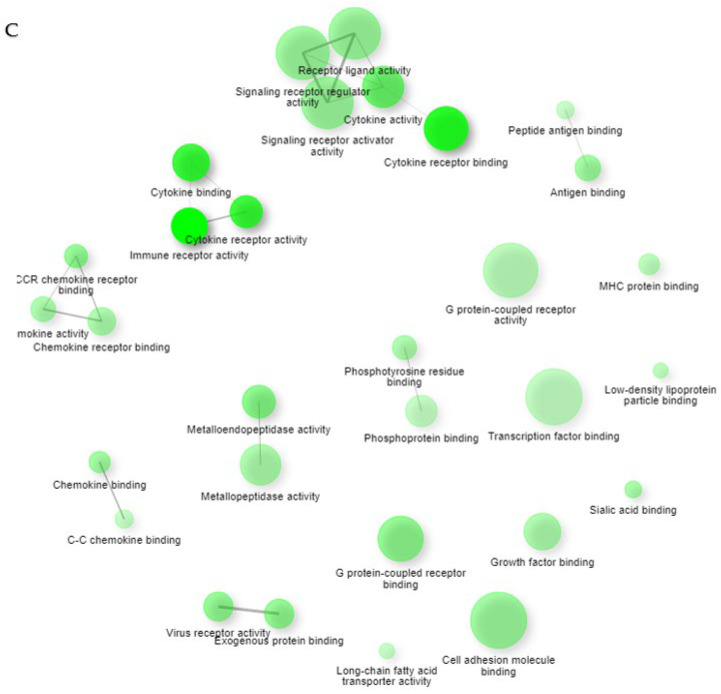
Pathway analysis with the GO molecular function with DEGs. ECRS-Ctrl (**A**), nonECRS-Ctrl (**B**), and Asp-Ctrl (**C**) showed the existence of significant enrichment pathways. Two pathways (nodes) are connected if they share 30% or more genes. Green and red represents down and up regulated pathways, respectively. Darker nodes show more significantly enriched gene sets. Bigger nodes represent larger gene sets. Thicker edges represent more overlapping genes.

Pathway analysis with the GO molecular function with DEGs in ECRS-Ctrl, nonECRS-Ctrl, and Asp-Ctrl showed the existence of significant up and downregulated pathways (Figure 5A–C), but Asp-nonECRS and Asp-ECRS showed no significant up and downregulated pathways. 

To clarify the detailed differences in the pathology of ECRS-Ctrl, nonECRS-Ctrl, and Asp-Ctrl in the common KEGG pathways, we analyzed the upregulated DEGs using DAVID. Genes with disease-associated terms such as “cytokine–cytokine receptor interaction”, “hematopoietic cell lineage”, “chemokine signaling pathway”, “complement and coagulation cascade”, “osteoclast differentiation”, “Staphylococcus aureus infection”, and “viral protein interaction with cytokine and cytokine receptor” were commonly upregulated in all CRSwNP groups vs control (Table 1).

The term “cytokine–cytokine receptor interaction” was commonly high-ranked in terms of the gene count in all CRSwNP groups vs control. DEGs in “Cytokine–Cytokine Receptor Interaction” showed that CCL13, CCL18, CCL26, TNFRSF18, INHBB, IL1RL1, and IL2RA were more upregulated in ECRS-Ctrl than nonECRS-Ctrl; CCL2, CCL8, CCL20, CCR5, CXCL1, CXCL6, CXCR2, FAS, TNFRSF1B, TNFSF13B, CSF3, IL2RG, IL6R, IL20RB, and IL23A were more upregulated in nonECRS-Ctrl than ECRS-Ctrl (Table 2). The upregulated genes in Asp-Ctrl were similar to the DEGs in ECRS-Ctrl and nonECRS-Ctrl. (Table 2). 

The KEGG pathway analysis with the downregulated DEGs of ECRS-Ctrl, nonECRS-Ctrl, and Asp-Ctrl also revealed that genes associated with the terms “metabolic pathways”, “salivary secretion”, “mucin type O-glycan biosynthesis”, and “glycine, serine and threonine metabolism” were commonly downregulated (Table 3). The gene count of “metabolic pathways” showed the highest count among the all CRSwNP groups vs Ctrl, and the gene counts were widely distributed as ECRS-Ctrl of 55, nonECRS-Ctrl of 36, and Asp-Ctrl of 65.

### 2.2. Segregation All CRSwNPs into Two Types of Clusters by Hierarchic Cluster Analysis 

**Figure 6:**
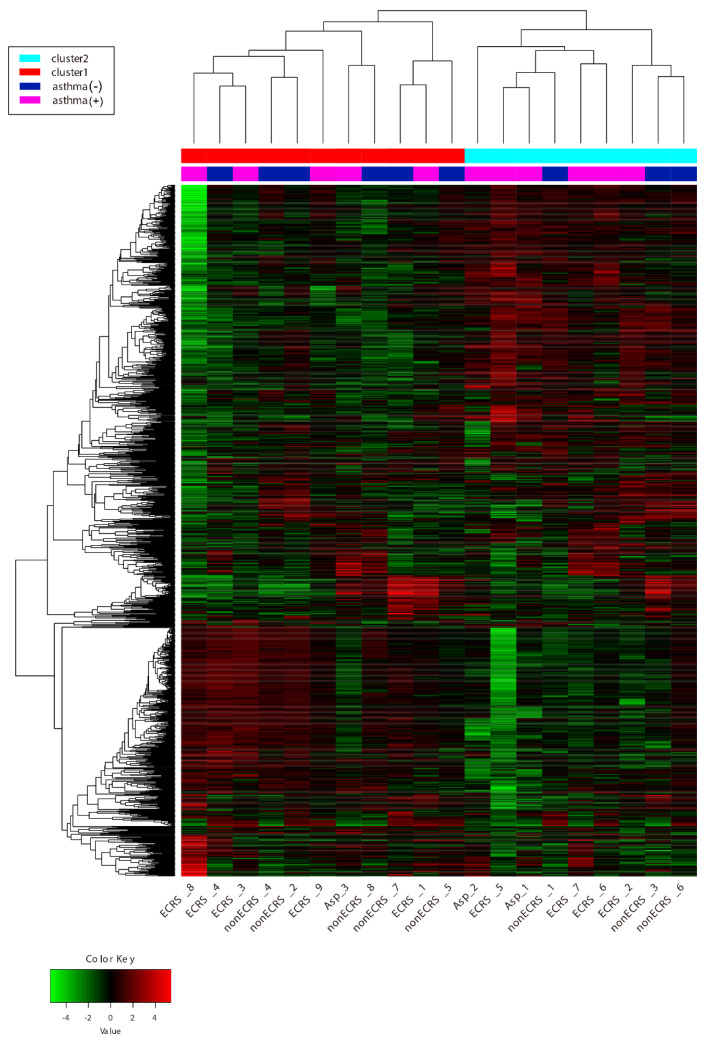
Hierarchic cluster analysis with all CRSwNPs segregated into two clusters (cluster1 and 2) among these CRSwNPs. Segregation of two clusters and comorbid of asthma were not correlated.

Due to the close association and similar clusters between ECRS, nonECRS, and Asp, we segregated these CRSwNPs into two clusters (cluster1 and 2) using the hierarchical cluster analysis with all CRSwNPs (Figure 6). The two clusters were not correlated with the comorbid of asthma. Additionally, height, weight, BMI, sex, age, comorbid of olfactory disturbance, otitis media, smoking, blood eosinophil, total IgE, nasal polyp score, CT score, tissue eosinophil, JESREC score, Oral FeNO, and Nasal FeNO were not correlated with the segregation (data not shown).

**Figure 7:**
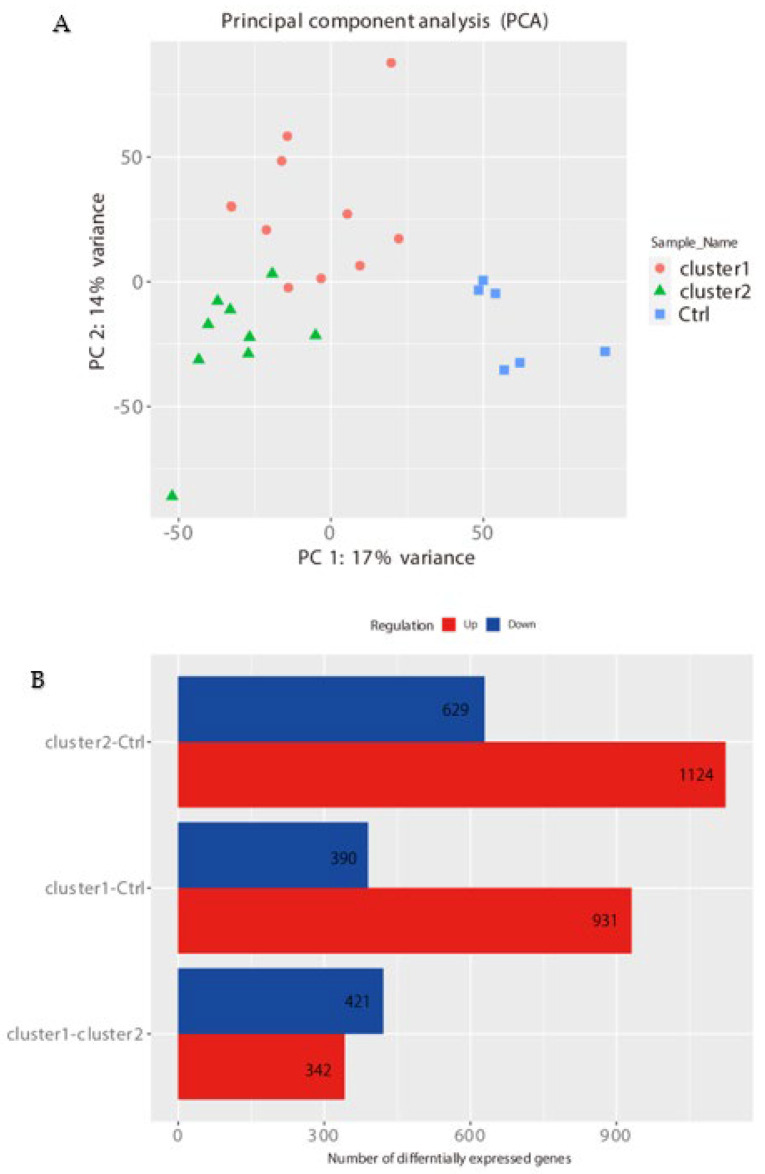
PCA analysis and DEGs of new clusters. (**A**) The new clusters showed clear separation from the control in PCA. (**B**) DEGs were identified not only for both cluster1-Ctrl and cluster2-Ctrl but also for cluster1-cluster2.

The new clusters and control showed clear separation in the PCA analysis and DEGs (Figure 7). 

The cluster-specific upregulated and downregulated DEGs (Table S1) were identified between cluster1 vs cluster2, and the KEGG pathway analysis by DAVID with upregulated DEGs revealed that the disease-associated terms “cytokine–cytokine receptor interaction” was commonly highly ranked between cluster1 vs control (cluster1-Ctrl) and cluster2 vs control (cluster2-Ctrl) (Table S2). 

**Figure 8:**
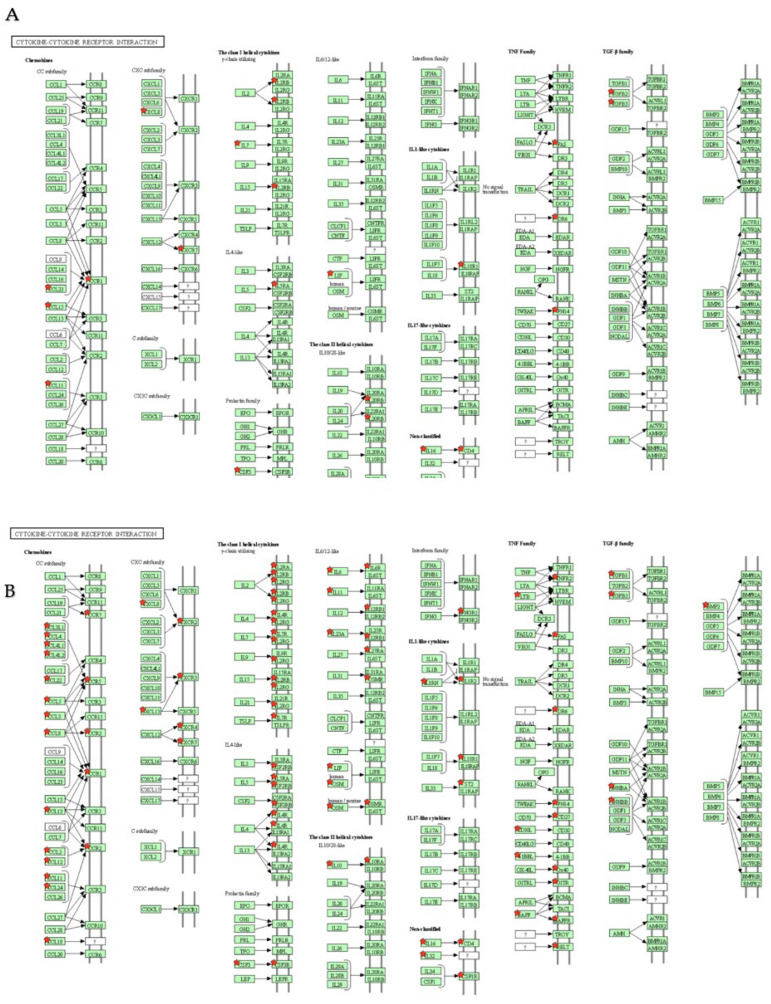
KEGG pathway analysis in “cytokine–cytokine receptor interaction” by DAVID for upregulated DEGs. Red stars represent the upregulated DEGs. Compared to cluster1-Ctrl (**A**) and cluster2-Ctrl (**B**), cluster2 was more upregulated for genes of the CC subfamily, CXC subfamily, gamma-chain utilizing, IL-4-like, IL6/12-like, IL-1-like cytokine, and TNF family.

Compared to cluster1-Ctrl and cluster2-Ctrl, cluster2 was more upregulated in genes of CC subfamily, CXC subfamily, gamma-chain utilizing, IL-4-like, IL6/12-like, IL-1-like cytokine, and TNF families associated with “cytokine–cytokine receptor interaction” (Figure 8).

## 3. Discussion

CRS has been categorized using the pathological molecular evidence and syndromic phenotypes, and currently it is believed that strong pathological associations between endotype and phenotypes would be the key factors to resolve the clinical problems of CRS therapies [3]. ECRS involving Asp is known as the most difficult-to-treat type of CRSwNP because of the easy relapse of NPs after sinus surgery and resistance to medical therapy except for oral systemic corticosteroids; nonECRS is known as an easy-to-treat type of CRSwNP with good response to ESS and ordinal macrolide therapy [10]. The pathological differences between ECRS and nonECRS were recognized by the type of inflammation: whether type 2-dependent with eosinophil infiltration or type 1-dependent. 

Currently, the criteria for categorizing ECRS have been readily accepted, especially in terms of the disease severity [11,12,13]. To detect clear differences in the type of inflammation and other factors, we performed the transcriptome analysis for ECRS, nonECRS, and Asp. The PCA and heatmap of the correlation matrix showed clear segregation between the control group and the others, but the others were not segregated. Additionally, hierarchical cluster analysis of the DEGs revealed no distinct segregation among the three CRSwNP groups, and some ECRS and nonECRS patients were segregated in a similar cluster as a control group. These results suggested that CRSwNP was widely distributed, but endotype separation using the current criteria for ECRS, which mainly use a count of eosinophil infiltration in NP and blood eosinophil count or positive of comorbid asthma and/or AIA, could not well-segregate CRSwNP into the pathological endotypes. Furthermore, these results also suggested that new criteria for the segregation of CRSwNPs to enhance the pathological endotypes should be proposed to analyze the effectiveness of various therapies.

In our study, a difference in significant enrichment pathways among the CRSwNP groups could not be detected using the DEGs because of no or a low number of DEGs. However, a difference in category-oriented significant enrichment pathways, which came from the comparison between each CRSwNP groups vs the control, could be identified, and “cytokine–cytokine receptor interaction” was mainly upregulated. Additionally, the DEGs associated with “cytokine–cytokine receptor interaction” among the three comparison groups were widely distributed, and the upregulated genes of *CCL13* and *CCL26* (eotaxin-3) in ECRS and Asp and *CCL18* in ECRS were identified in either ECRS or Asp compared with nonECRS. In addition, *CCL11*(eotaxin) was upregulated in ECRS and nonECRS, and *CCL24* (eotaxin-2) was not identified in any CRSwNPs. Previous reports mentioned that type 2 inflammatory transcript expression of *CCL13*, *CCL18*, and *CCL26* were upregulated in the type 2 endotype [7] and ECRS compared with nonECRS [8]. It is known that *CCL11*, *CCL13*, and *CCL26* recruit eosinophils, basophils, mast cells, and *CD4*(+) T helper 2 cells [14], and *CCL18* recruits naive T cells, B cells, immature dendritic cells, and activate fibroblasts [15]. *CCL18* is thought to be involved in T(H)2-related inflammatory diseases, including asthma and atopic dermatitis, and the number of *CCL18*(+) cells was significantly increased within NPs [15]. Production of *CCL26* is induced by IL4/13 in epithelial cells [16], and *CCL26* induces not only eosinophil infiltration but also NK cell migration, which contributes to trafficking NK cells to the allergic upper airway mediated by *CX3CL1* and the tyrosine kinase pathway [17]. Higher expression of type 2 inflammatory molecules (*CCL13, IGHE, CCL18, CCL23, CCR3,* and *CLC*) along with lower levels of *LACRT, PPDPFL, DES, C6, MUC5B,* and *SCGB3A1* are related to a stronger clinical response to glucocorticoids [18]. In terms of the above-mentioned evidence, these differences would explain the difference of each phenotype as to whether they mainly have type 2 inflammation or not. From the upregulation of these gene expressions, we concluded that ECRS have higher type 2 inflammation with the upregulation of *CCL13*, *CCL18*, and *CCL26* than nonECRS, and eosinophil infiltration in ECRS would be mainly induced by *CCL13* and *CCL26* rather than *CCL11* and *CCL24*.

Furthermore, the upregulated expression of *TNFRSF18*, *IL1RL1*, and *IL2RA* in ECRS and Asp, *INHBB* in ECRS, and *TNFRSF10D*, *IL6*, *IL11*, and *IL31RA* in Asp is also associated with “cytokine–cytokine receptor interaction”. *IL1RL1* is the receptor of *IL33*, which induces T helper 2-type inflammatory responses. *IL1RL1*-positive cells in the inflammatory cells of the subepithelial layer are significantly higher, and the expression of *IL1RL1* in polyps is significantly increased in the ECRS compared with controls. Many *IL1RA1*-positive eosinophils are observed only in the mucosa of ECRS but not of nonECRS [19]. Therefore, overexpression of *IL1RA1* in ECRS and Asp suggested that higher type 2 inflammation would be induced by *IL33* and *IL1RA1* with *CD4*(+) T helper 2 cells recruited by *CCL11*, *CCL13*, and *CCL26*. Furthermore, *TNFRSF10D* is expressed by macrophages/monocytes and promotes eosinophil survival via inhibiting the spontaneous apoptosis [20]. *IL11* is selectively expressed in eosinophils and epithelial cells in patients with moderate and severe asthma, and correlates directly with disease severity. *IL11* also causes nodular mononuclear infiltrates [21]. *IL31* and *IL31RA* induce *MUC5AC* gene expression in human airway epithelial cells and play important roles in mucus overproduction [22]. These findings suggested that eosinophil accumulation caused by prolonged survival of eosinophil with *TNFRSF10D*, nodular mononuclear infiltration with *IL11*, and mucus overexpression with *MUC5AC* via *IL31RA* would be the characteristic features of Asp. In the same way as *IL11* enhances the disease severity in asthma, overexpression of *IL6* and *IL11* in Asp may reflect the severity of IL6-mediated inflammation and type 2 inflammation enhancing the disease severity in CRS.

Overexpressions of *TNFRSF18*, *IL2RA*, and *INHBB* in either ECRS or Asp groups may also reflect the disease severity and pathology of the CRSwNP phenotype via interference of cytokine–cytokine receptor interaction despite poor reports of pathological analyses between the overexpressions of these genes and the phenotype of ECRS and/or Asp.

However, the KEGG pathway analysis for the downregulated DEGs of ECRS-Ctrl, nonECRS-Ctrl, and Asp-Ctrl also revealed that “metabolic pathways” would be another factor for the CRSwNP phenotype. The gene count differences of “metabolic pathways”, namely ECRS-Ctrl of 55, nonECRS-Ctrl of 36, and Asp-Ctrl of 65, also pave the way to elucidate the differences in each phenotype associated with disease severity.

Hierarchical cluster analysis using the DEGs revealed no distinct segregation among the three CRSwNP groups, but it did suggest that these CRSwNPs segregate into two clusters in terms of the similarity of up and downregulated DEGs. Two clusters (cluster 1 and 2) among all CRSwNPs, after modifying a hierarchical cluster analysis, showed clear separation in the PCA analysis. The cluster-specific upregulated DEGs and the KEGG pathway analysis by DAVID of two clusters and the control group revealed that the disease-associated term “cytokine–cytokine receptor interaction” was commonly highly ranked. Compared to cluster1-Ctrl and cluster2-Ctrl in the DEGs of “cytokine–cytokine receptor interaction”, cluster2 was more upregulated in the genes of the CC subfamily and CXC subfamily in “chemokines”, gamma-chain utilizing, and IL-4 like and IL-6/12-like genes in “the class I helical cytokines”, “IL1-like cytokines”, and “TNF family”. In the IL4-like genes in “the class I helical cytokines”, a comparison between cluster1-Ctrl and cluster2-Ctrl revealed that cluster2 was more upregulated in the expression of *CSF2RB* and *IL4R* than cluster1. These genes are deeply associated with type 2 inflammation via *IL4*, *IL5*, and *IL13* signaling [23,24]. Previously, dupilumab, which is a dual inhibitor of *IL4* and *IL13* signaling and controls the type 2 inflammation, was shown to decrease several symptoms of CRSwNP despite the diversity in the degree of eosinophilic infiltration of sinonasal tissue by classifying CRSwNP into the categories from nonECRS to severe ECRS [25]. Given the result of the effectiveness of dupilumab against all CRSwNP, all CRSwNP groups should have type 2 inflammation, which could differ in degree regardless of the criteria for ECRS. The upregulated expression of *CSF2RB* and *IL4R* in cluster2 is expected to be strongly associated with the effect of dupilumab by blocking this signaling. In addition, the segregation of DEGs into cluster1 and cluster2 will elucidate the broad effectiveness of dupilumab in all CRSwNPs in spite of the phenotypes of nonECRS, which are suggested to be deeply associated with type 1 inflammation. The segregation of DEGs into cluster1 and 2 will become the criteria for true endotype segregation, and further studies are needed to categorize CRSwNP enhancement by/of *IL4*, *IL5*, and *IL13* signaling.

There are some limitations to our study. First, due to the small sample size in Asp, we could not demonstrate more clear differences in DEGs between ECRS and nonECRS, and the tendency as to whether Asp could be classified as more cluster1 or 2. Second, this study only included Japanese patients; hence, caution should be taken when extrapolating our results to other ethnic groups. Third, we could not measure expression protein levels. A previous report mentioned that the protein expression levels of *CCL13* and *CCL26* were minimal or unchanged compared with the control despite the overexpression of these mRNAs [26]. Further studies are needed to analyze the correlation among the CRS phenotype, protein expression levels, and transcriptional overexpression of *CCL13* and *CCL26.* Additionally, some protein expression levels may also be minimal or unchanged despite transcriptomic overexpression. Further studies are necessary to explore the correlation between the DEGs detected in BRB-seq and protein expression levels.

## 4. Materials and Methods

### 4.1. Patient Recruitment

Patients with or without CRS, who underwent endoscopic sinus surgery in Hiroshima Medical University Hospital between October 2016 and October 2021, were enrolled in this study. The diagnosis of CRS was based on computed tomography (CT) scanning, patient history, clinical symptoms, and endoscopic findings. The inclusion criteria for CRSwNP were as follows: treatment without oral/nasal steroids within 4 weeks before surgery, no improvement in continuous nasal drip, post-nasal drip, and nasal congestion after medical treatment including low-dose macrolide therapy. Patients with CRSwNP were clinically diagnosed as ECRS or nonECRS based on the diagnostic criteria of the JESREC study [4]. The diagnostic criteria for ECRS (JESREC score) include (1) side affected: both sides, 3 points; (2) with nasal polyps, 2 points; (3) CT changes: ethmoid/maxillary ≥ 1, 2 points; (4) peripheral blood eosinophil count (%): 2 < and ≤ 5%, 4 points; 5 < and ≤ 10%, 8 points; 10%<, 10 points. Eosinophil infiltration into the NPs was diagnosed by calculating the mean cell count of the 3 most dense areas of eosinophils in the hematoxylin and eosin (HE) stained sections under ×400 magnification. A diagnosis of ECRS was made by both the total JESREC score of 11 points or higher and eosinophil infiltration into the NPs of 70 or more [4]. The group of ECRS with comorbid aspirin intolerant asthma was independently classified as Asp (N = 3), and the others were classified as ECRS (N = 9) and nonECRS (N = 8). Control patients (Ctrl) (N = 6) were also diagnosed based on the anatomical abnormalities but showed no inflammatory mucosal change or bacterial infection around the uncinate process. The exclusion criteria for CRSwNP were as follows: treatment with oral/nasal steroids within 4 weeks before surgery, fungal/allergic fungal rhinosinusitis, and primary ciliary dyskinesia. Demographics and clinical characteristics were obtained from the medical records. The detailed patient demography is shown in Table S3.

### 4.2. RNA-Seq Using BRB-Seq

BRB-seq [9] was performed for library preparation with the following modifications. Barcoded oligo-dT-based primer (5′-GCCGGTAATACGACTCACTATAGGGAGTTCTACAGTCCGACGATCNNNNNNNNNNCCCCCCCCCTTTTTTTTTTTTTTTTTTTTTTTTV -3′; 10bp “N” = UMI, 9bp “C” = cell barcode) was used for single-stranded synthesis and Second Strand Synthesis Module (New England Biolabs, Ipswich, MA, USA, #E6111) was used for double-stranded cDNA synthesis. In-house MEDS-B Tn5 transposase [27,28] was used for tagmentation, and libraries were amplified for 10 cycles of PCR using Phusion High-Fidelity DNA Polymerase (Thermo Scientific, Waltham, MA, USA, #M0530) with the following primers (5′ -AATGATACGGCGACCACCGAGATCTACACindexGTTCAGAGTTCTACAGTCCGA-3′,5′-CAAGCAGAAGACGGCATACGAGATindexGTCTCGTGGGCTCGGAGATGT-3′). An Illumina NovaSeq6000 (Illumina, San Diego, CA, USA) was used to obtain 15 bp of barcode read (Read1) and 81 bp of insert read (Read2).

### 4.3. Data Processing of BRB-Seq

Read1 (barcode read) was extracted by using UMI-tools (ver.1.1.1) with the command “umi_tools extract -I read1.fastq --read2-in=read2.fastq --bc-pattern=NNNNNNNNNNCCCCCCCCC --read2-stdout”. Adaptor sequences and low-quality sequences were removed and read length below 20 bp were discarded using Trim Galore (ver.0.6.7). Reads were mapped to the GRCh38 reference using HISAT2 (ver.2.2.1). Read counts for each gene were obtained by feature Counts (ver.2.0.1), and UMI duplication was removed by UMI-tools with the command “umi_tools count --method=unique --per-gene --per-cell --gene-tag=XT”. Normalized read count value was obtained by using DESeq2 (ver.1.34.0) (Table S4). Gene-level expression data (read counts) were processed by using the web portal for integrated differential expression and pathway analysis (iDEP.95; http://bioinformatics.sdstate.edu/idep, accessed on 25 April 2022). iDEP.95 was used for principal component analysis (PCA), hierarchical clustering with a heatmap, identification of differentially expressed genes (DEGs), and pathway analysis. DEGs were extracted with an FDR cutoff of 0.1 and min-fold change of 2. Pathway analysis was performed using GAGE with the genesets of GO Molecular Function. Geneset size was set at minimum 5 and maximum 2000, and pathway significance cutoff (FDR) was set at 0.2. To ascertain the functions of the differential expressed mRNAs, Kyoto Encyclopedia of Genes and Genomes (KEGG) pathway analysis was used with the Database for Annotation, Visualization, and Integrated Discovery (DAVID; https://david.ncifcrf.gov/, accessed on 25 April 2022).

## 5. Conclusions

This study revealed and shed light on the future gene expression targets for endotype categorization. Additionally, this study also revealed that BRB-seq was useful to elucidate the differences in CRSwNP. BRB-seq allowed us to perform a transcriptome analysis for as many as 26 patients because of the low cost of the sequencing. A greater number of patients could help us to segregate into new clusters and elucidate the differences between them. This method may provide a valuable tool for further studies to analyze the differences in CRSwNPs in terms of the phenotype and endotype categorization.

## Figures and Tables

**Table 1:** KEGG pathways using the upregulated DEGs into DAVID. Genes with the disease-associated terms “cytokine–cytokine receptor interaction”, “hematopoietic cell lineage”, “chemokine signaling pathway”, “complement and coagulation cascade”, “osteoclast differentiation”, “Staphylococcus aureus infection”, and “viral protein interaction with cytokine and cytokine receptor” were commonly upregulated in all CRSwNP groups vs control.

Comparison	Term	Count	%	*p*-Value	Benjamini
ECRS- Ctrl UP	hsa05200:Pathways in cancer	35	5.2	6.7 × 10^−4^	2.6 × 10^−2^
	hsa04060:Cytokine–cytokine receptor interaction	25	3.7	1.4 × 10^−4^	1.0 × 10^−2^
	hsa04080:Neuroactive ligand–receptor interaction	22	3.2	1.5 × 10^−2^	2.5 × 10^−1^
	hsa04151:PI3K-Akt signaling pathway	19	2.8	8.5 × 10^−2^	7.3 × 10^−1^
	hsa05166:Human T-cell leukemia virus 1 infection	18	2.7	2.6 × 10^−3^	7.0 × 10^−2^
	hsa04020:Calcium signaling pathway	18	2.7	5.6 × 10^−3^	1.0 × 10^−1^
	hsa04640:Hematopoietic cell lineage	17	2.5	3.8 × 10^−7^	1.0 × 10^−4^
	hsa04145:Phagosome	16	2.4	3.5 × 10^−4^	1.9 × 10^−2^
	hsa04062:Chemokine signaling pathway	16	2.4	3.7 × 10^−3^	7.8 × 10^−2^
	hsa04610:Complement and coagulation cascades	15	2.2	1.6 × 10^−6^	2.2 × 10^−4^
	hsa04380:Osteoclast differentiation	14	2.1	6.5 × 10^−4^	2.6 × 10^−2^
	hsa05202:Transcriptional misregulation in cancer	14	2.1	2.1 × 10^−2^	2.8 × 10^−1^
	hsa05150:Staphylococcus aureus infection	13	1.9	1.5 × 10^−4^	1.0 × 10^−2^
	hsa05152:Tuberculosis	13	1.9	2.8 × 10^−2^	3.4 × 10^−1^
	hsa04061:Viral protein interaction with cytokine and cytokine receptor	12	1.8	8.7 × 10^−4^	3.0 × 10^−2^
	hsa05142:Chagas disease	11	1.6	3.5 × 10^−3^	7.8 × 10^−2^
	hsa05146:Amoebiasis	11	1.6	3.5 × 10^−3^	7.8 × 10^−2^
	hsa04514:Cell adhesion molecules	11	1.6	4.2 × 10^−2^	4.2 × 10^−1^
	hsa05140:Leishmaniasis	10	1.5	1.6 × 10^−3^	5.0 × 10^−2^
	hsa04659:Th17 cell differentiation	10	1.5	1.5 × 10^−2^	2.5 × 10^−1^
	hsa04658:Th1 and Th2 cell differentiation	9	1.3	1.7 × 10^−2^	2.6 × 10^−1^
	hsa04611:Platelet activation	9	1.3	7.7 × 10^−2^	6.8 × 10^−1^
	hsa04512:ECM-receptor interaction	8	1.2	3.8 × 10^−2^	4.0 × 10^−1^
	hsa04750:Inflammatory mediator regulation of TRP channels	8	1.2	6.2 × 10^−2^	5.6 × 10^−1^
	hsa04066:HIF-1 signaling pathway	8	1.2	9.6 × 10^−2^	8.0 × 10^−1^
	hsa04978:Mineral absorption	7	1.0	2.0 × 10^−2^	2.8 × 10^−1^
	hsa00590:Arachidonic acid metabolism	7	1.0	2.2 × 10^−2^	2.8 × 10^−1^
	hsa05321:Inflammatory bowel disease	7	1.0	2.8 × 10^−2^	3.4 × 10^−1^
	hsa04664:Fc epsilon RI signaling pathway	7	1.0	3.5 × 10^−2^	3.8 × 10^−1^
	hsa05133:Pertussis	7	1.0	5.5 × 10^−2^	5.3 × 10^−1^
	hsa05310:Asthma	6	0.9	4.5 × 10^−3^	8.8 × 10^−2^
	hsa05219:Bladder cancer	5	0.7	5.8 × 10^−2^	5.5 × 10^−1^
	hsa00532:Glycosaminoglycan biosynthesis—chondroitin sulfate/dermatan sulfate	4	0.6	3.3 × 10^−2^	3.8 × 10^−1^
nonECRS-Ctrl UP	hsa04060:Cytokine–cytokine receptor interaction	33	4.7	1.5 × 10^−8^	2.0 × 10^−6^
	hsa05200:Pathways in cancer	33	4.7	2.4 × 10^−3^	2.7 × 10^−2^
	hsa04080:Neuroactive ligand–receptor interaction	23	3.3	7.8 × 10^−3^	7.6 × 10^−2^
	hsa04062:Chemokine signaling pathway	22	3.1	4.5 × 10^−6^	1.7 × 10^−4^
	hsa05166:Human T-cell leukemia virus 1 infection	21	3.0	1.3 × 10^−4^	2.2 × 10^−3^
	hsa04061:Viral protein interaction with cytokine and cytokine receptor	19	2.7	1.2 × 10^−8^	2.0 × 10^−6^
	hsa05150:Staphylococcus aureus infection	18	2.6	3.9 × 10^−8^	3.4 × 10^−6^
	hsa05152:Tuberculosis	18	2.6	2.3 × 10^−4^	3.6 × 10^−3^
	hsa04640:Hematopoietic cell lineage	17	2.4	3.7 × 10^−7^	2.4 × 10^−5^
	hsa05417:Lipid and atherosclerosis	17	2.4	4.3 × 10^−3^	4.5 × 10^−2^
	hsa05323:Rheumatoid arthritis	16	2.3	8.8 × 10^−7^	4.6 × 10^−5^
	hsa04145:Phagosome	16	2.3	3.4 × 10^−4^	4.7 × 10^−3^
	hsa05171:Coronavirus disease—COVID-19	16	2.3	1.9 × 10^−2^	1.3 × 10^−1^
	hsa04659:Th17 cell differentiation	15	2.1	2.8 × 10^−5^	7.3 × 10^−4^
	hsa04380:Osteoclast differentiation	15	2.1	1.8 × 10^−4^	3.0 × 10^−3^
	hsa05167:Kaposi sarcoma-associated herpesvirus infection	15	2.1	9.6 × 10^−3^	8.5 × 10^−2^
	hsa05169:Epstein–Barr virus infection	15	2.1	1.3 × 10^−2^	1.1 × 10^−1^
	hsa05163:Human cytomegalovirus infection	15	2.1	3.1 × 10^−2^	1.9 × 10^−1^
	hsa05321:Inflammatory bowel disease	13	1.9	2.6 × 10^−6^	1.1 × 10^−4^
	hsa05140:Leishmaniasis	13	1.9	1.6 × 10^−5^	4.7 × 10^−4^
	hsa04658:Th1 and Th2 cell differentiation	13	1.9	9.7 × 10^−5^	2.0 × 10^−3^
	hsa04630:JAK-STAT signaling pathway	13	1.9	1.3 × 10^−2^	1.1 × 10^−1^
	hsa05164:Influenza A	13	1.9	1.9 × 10^−2^	1.3 × 10^−1^
	hsa05142:Chagas disease	12	1.7	9.9 × 10^−4^	1.2 × 10^−2^
	hsa04668:TNF signaling pathway	12	1.7	2.1 × 10^−3^	2.5 × 10^−2^
	hsa05322:Systemic lupus erythematosus	12	1.7	9.3 × 10^−3^	8.5 × 10^−2^
	hsa04936:Alcoholic liver disease	12	1.7	1.3 × 10^−2^	1.1 × 10^−1^
	hsa04514:Cell adhesion molecules	12	1.7	1.8 × 10^−2^	1.3 × 10^−1^
	hsa05202:Transcriptional misregulation in cancer	12	1.7	8.3 × 10^−2^	4.7 × 10^−1^
	hsa04610:Complement and coagulation cascades	11	1.6	8.5 × 10^−4^	1.1 × 10^−2^
	hsa04650:Natural killer cell mediated cytotoxicity	11	1.6	1.5 × 10^−2^	1.1 × 10^−1^
	hsa05161:Hepatitis B	11	1.6	6.5 × 10^−2^	3.9 × 10^−1^
	hsa04657:IL-17 signaling pathway	10	1.4	6.3 × 10^−3^	6.4 × 10^−2^
	hsa05145:Toxoplasmosis	10	1.4	1.9 × 10^−2^	1.3 × 10^−1^
	hsa05310:Asthma	9	1.3	9.3 × 10^−6^	3.1 × 10^−4^
	hsa05330:Allograft rejection	9	1.3	4.6 × 10^−5^	1.1 × 10^−3^
	hsa05332:Graft-versus-host disease	9	1.3	9.9 × 10^−5^	2.0 × 10^−3^
	hsa04940:Type I diabetes mellitus	9	1.3	1.2 × 10^−4^	2.2 × 10^−3^
	hsa04672:Intestinal immune network for IgA production	9	1.3	3.0 × 10^−4^	4.5 × 10^−3^
	hsa05320:Autoimmune thyroid disease	9	1.3	5.3 × 10^−4^	7.0 × 10^−3^
	hsa04662:B cell receptor signaling pathway	9	1.3	8.7 × 10^−3^	8.2 × 10^−2^
	hsa04612:Antigen processing and presentation	8	1.1	2.1 × 10^−2^	1.4 × 10^−1^
	hsa05146:Amoebiasis	8	1.1	7.2 × 10^−2^	4.2 × 10^−1^
	hsa04625:C-type lectin receptor signaling pathway	8	1.1	7.8 × 10^−2^	4.5 × 10^−1^
	hsa05416:Viral myocarditis	7	1.0	2.0 × 10^−2^	1.3 × 10^−1^
	hsa05133:Pertussis	7	1.0	5.4 × 10^−2^	3.3 × 10^−1^
	hsa05219:Bladder cancer	6	0.9	1.5 × 10^−2^	1.1 × 10^−1^
	hsa04664:Fc epsilon RI signaling pathway	6	0.9	9.5 × 10^−2^	5.2 × 10^−1^
Asp-Ctrl UP	hsa05200:Pathways in cancer	24	6.3	6.4 × 10^−3^	1.3 × 10^−1^
	hsa04151:PI3K-Akt signaling pathway	20	5.2	1.3 × 10^−3^	4.5 × 10^−2^
	hsa04060:Cytokine–cytokine receptor interaction	19	5.0	3.9 × 10^−4^	1.9 × 10^−2^
	hsa04062:Chemokine signaling pathway	16	4.2	8.4 × 10^−5^	1.0 × 10^−2^
	hsa05202:Transcriptional misregulation in cancer	15	3.9	3.0 × 10^−4^	1.8 × 10^−2^
	hsa04630:JAK-STAT signaling pathway	14	3.7	1.9 × 10^−4^	1.6 × 10^−2^
	hsa05206:MicroRNAs in cancer	14	3.7	4.6 × 10^−2^	4.1 × 10^−1^
	hsa05205:Proteoglycans in cancer	13	3.4	5.1 × 10^−3^	1.3 × 10^−1^
	hsa04610:Complement and coagulation cascades	12	3.1	7.4 × 10^−6^	1.8 × 10^−3^
	hsa05166:Human T-cell leukemia virus 1 infection	12	3.1	2.3 × 10^−2^	3.0 × 10^−1^
	hsa04020:Calcium signaling pathway	12	3.1	3.7 × 10^−2^	3.5 × 10^−1^
	hsa05167:Kaposi sarcoma-associated herpesvirus infection	11	2.9	2.3 × 10^−2^	3.0 × 10^−1^
	hsa05132:Salmonella infection	11	2.9	9.3 × 10^−2^	6.2 × 10^−1^
	hsa04144:Endocytosis	11	2.9	9.4 × 10^−2^	6.2 × 10^−1^
	hsa04380:Osteoclast differentiation	10	2.6	4.6 × 10^−3^	1.2 × 10^−1^
	hsa04015:Rap1 signaling pathway	10	2.6	7.9 × 10^−2^	6.0 × 10^−1^
	hsa04625:C-type lectin receptor signaling pathway	9	2.3	4.3 × 10^−3^	1.2 × 10^−1^
	hsa04066:HIF−1 signaling pathway	9	2.3	5.7 × 10^−3^	1.3 × 10^−1^
	hsa04061:Viral protein interaction with cytokine and cytokine receptor	8	2.1	1.2 × 10^−2^	2.3 × 10^−1^
	hsa05142:Chagas disease	8	2.1	1.3 × 10^−2^	2.4 × 10^−1^
	hsa04064:NF-kappa B signaling pathway	8	2.1	1.5 × 10^−2^	2.4 × 10^−1^
	hsa04668:TNF signaling pathway	8	2.1	2.2 × 10^−2^	3.0 × 10^−1^
	hsa04650:Natural killer cell mediated cytotoxicity	8	2.1	3.8 × 10^−2^	3.5 × 10^−1^
	hsa05135:Yersinia infection	8	2.1	5.5 × 10^−2^	4.8 × 10^−1^
	hsa04072:Phospholipase D signaling pathway	8	2.1	7.6 × 10^−2^	6.0 × 10^−1^
	hsa04218:Cellular senescence	8	2.1	9.4 × 10^−2^	6.2 × 10^−1^
	hsa05219:Bladder cancer	7	1.8	4.8 × 10^−4^	2.0 × 10^−2^
	hsa04657:IL-17 signaling pathway	7	1.8	2.9 × 10^−2^	3.5 × 10^−1^
	hsa05150:Staphylococcus aureus infection	7	1.8	3.2 × 10^−2^	3.5 × 10^−1^
	hsa04666:Fc gamma R-mediated phagocytosis	7	1.8	3.4 × 10^−2^	3.5 × 10^−1^
	hsa04640:Hematopoietic cell lineage	7	1.8	3.7 × 10^−2^	3.5 × 10^−1^
	hsa04115:p53 signaling pathway	6	1.6	3.5 × 10^−2^	3.5 × 10^−1^
	hsa04012:ErbB signaling pathway	6	1.6	6.0 × 10^−2^	5.1 × 10^−1^
	hsa05210:Colorectal cancer	6	1.6	6.3 × 10^−2^	5.1 × 10^−1^
	hsa04216:Ferroptosis	5	1.3	1.8 × 10^−2^	2.8 × 10^−1^
	hsa05211:Renal cell carcinoma	5	1.3	9.3 × 10^−2^	6.2 × 10^−1^
	hsa05230:Central carbon metabolism in cancer	5	1.3	9.6 × 10^−2^	6.2 × 10^−1^
	hsa05120:Epithelial cell signaling in Helicobacter pylori infection	5	1.3	9.6 × 10^−2^	6.2 × 10^−1^

**Table 2:** Upregulated genes for “cytokine–cytokine receptor interaction.” Upregulated DEGs were represented with 〇. *CCL13*, *CCL18*, *CCL26*, *TNFRSF18*, *INHBB*, *IL1RL1*, and *IL2RA* in ECRS-Ctrl were more upregulated compared to nonECRS-Ctrl, and *CCL2*, *CCL8*, *CCL20*, *CCR5*, *CXCL1*, *CXCL6*, *CXCR2*, *FAS*, *TNFRSF1B*, *TNFSF13B*, *CSF3*, *IL2RG*, *IL20RB*, and *IL23A* in nonECRS-Ctrl were more upregulated compared to ECRS-Ctrl. The upregulated genes in Asp-Ctrl were similar to the DEGs of ECRS-Ctrl and nonECRS-Ctrl, but *TNFRSF10D*, *IL6*, *IL11*, *IL31RA* were only upregulated in Asp-Ctrl compared with the DEGs of ECRS-Ctrl and nonECRS-Ctrl.

	Genes	ECRS-Ctrl	NonECRS-Ctrl	Asp-Ctrl
Upregulated DEGs	CCL2		〇	
	CCL8		〇	
	CCL11	〇	〇	
	CCL13	〇		〇
	CCL15	〇	〇	
	CCL18	〇		
	CCL20		〇	
	CCL26	〇		〇
	CCR1	〇	〇	〇
	CCR5		〇	
	CXCL1		〇	
	CXCL6		〇	
	CXCL8	〇	〇	〇
	CXCR2		〇	
	CD4	〇	〇	
	FAS		〇	
	LIF	〇	〇	〇
	TNFRSF1B		〇	
	TNFRSF10D			〇
	TNFRSF12A	〇	〇	〇
	TNFRSF18	〇		〇
	TNFRSF21	〇	〇	〇
	TNFSF13B		〇	
	ACKR3	〇	〇	
	CSF1R	〇	〇	
	CSF2RB	〇	〇	
	CSF3		〇	〇
	INHBB	〇		
	IL1RL1	〇		〇
	IL2RA	〇		〇
	IL2RB	〇	〇	
	IL2RG		〇	
	IL4R	〇	〇	
	IL5RA	〇	〇	〇
	IL6			〇
	IL6R		〇	
	IL11			〇
	IL16	〇	〇	
	IL18R1	〇	〇	〇
	IL20RB		〇	
	IL23A		〇	〇
	IL31RA			〇
	OSMR	〇	〇	〇
	TGFB3	〇	〇	

**Table 3:** KEGG pathways using the downregulated DEGs into the DAVID. Genes associated with “metabolic pathways”, “salivary secretion”, “mucin type O-glycan biosynthesis”, and “glycine, serine and threonine metabolism” were commonly downregulated. The gene count for “metabolic pathways” was the highest among all the CRSwNP groups vs control, and the gene counts were widely distributed as ECRS-Ctrl of 55, nonECRS-Ctrl of 36, and Asp-Ctrl of 65.

Comparison	Term	Count	%	*p*-Value	Benjamini
ECRS-Ctrl down	hsa01100:Metabolic pathways	55	11.5	5.6 × 10^−3^	2.4 × 10^−1^
	hsa04970:Salivary secretion	15	3.1	7.6 × 10^−8^	1.9 × 10^−5^
	hsa04024:cAMP signaling pathway	12	2.5	2.5 × 10^−2^	8.1 × 10^−1^
	hsa04972:Pancreatic secretion	11	2.3	2.5 × 10^−4^	3.2 × 10^−2^
	hsa04550:Signaling pathways regulating pluripotency of stem cells	11	2.3	3.4 × 10^−3^	1.8 × 10^−1^
	hsa04020:Calcium signaling pathway	11	2.3	8.5 × 10^−2^	1.0
	hsa05215:Prostate cancer	9	1.9	3.2 × 10^−3^	1.8 × 10^−1^
	hsa00260:Glycine, serine and threonine metabolism	6	1.3	3.2 × 10^−3^	1.8 × 10^−1^
	hsa05412:Arrhythmogenic right ventricular cardiomyopathy	6	1.3	4.6 × 10^−2^	1.0
	hsa04911:Insulin secretion	6	1.3	6.7 × 10^−2^	1.0
	hsa04350:TGF-beta signaling pathway	6	1.3	9.0 × 10^−2^	1.0
	hsa00512:Mucin type O-glycan biosynthesis	5	1.0	1.3 × 10^−2^	4.6 × 10^−1^
	hsa00514:Other types of O-glycan biosynthesis	5	1.0	3.1 × 10^−2^	8.8 × 10^−1^
	hsa05031:Amphetamine addiction	5	1.0	9.8 × 10^−2^	1.0
	hsa00100:Steroid biosynthesis	3	0.6	9.0 × 10^−2^	1.0
nonECRS-Ctrl down	hsa01100:Metabolic pathways	36	11.6	7.1 × 10^−3^	3.9 × 10^−1^
	hsa04970:Salivary secretion	13	4.2	1.4 × 10^−8^	3.1 × 10^−6^
	hsa04972:Pancreatic secretion	10	3.2	2.4 × 10^−5^	2.6 × 10^−3^
	hsa04024:cAMP signaling pathway	10	3.2	6.6 × 10^−3^	3.9 × 10^−1^
	hsa04911:Insulin secretion	6	1.9	1.0 × 10^−2^	4.4 × 10^−1^
	hsa05231:Choline metabolism in cancer	5	1.6	6.3 × 10^−2^	1.0
	hsa00512:Mucin type O-glycan biosynthesis	4	1.3	1.7 × 10^−2^	5.9 × 10^−1^
	hsa05143:African trypanosomiasis	4	1.3	1.9 × 10^−2^	5.9 × 10^−1^
	hsa00260:Glycine, serine and threonine metabolism	4	1.3	2.3 × 10^−2^	6.3 × 10^−1^
	hsa00280:Valine, leucine and isoleucine degradation	4	1.3	3.7 × 10^−2^	9.0 × 10^−1^
	hsa05031:Amphetamine addiction	4	1.3	8.8 × 10^−2^	1.0
Asp-Ctrl down	hsa01100:Metabolic pathways	65	14.0	2.4 × 10^−6^	5.9 × 10^−4^
	hsa04970:Salivary secretion	12	2.6	1.3 × 10^−5^	1.7 × 10^−3^
	hsa01200:Carbon metabolism	8	1.7	2.1 × 10^−2^	7.5 × 10^−1^
	hsa00280:Valine, leucine and isoleucine degradation	7	1.5	9.7 × 10^−4^	8.0 × 10^−2^
	hsa04972:Pancreatic secretion	7	1.5	3.7 × 10^−2^	9.1 × 10^−1^
	hsa00514:Other types of O-glycan biosynthesis	6	1.3	5.3 × 10^−3^	3.1 × 10^−1^
	hsa00520:Amino sugar and nucleotide sugar metabolism	6	1.3	6.3 × 10^−3^	3.1 × 10^−1^
	hsa04976:Bile secretion	6	1.3	6.4 × 10^−2^	1.0
	hsa00564:Glycerophospholipid metabolism	6	1.3	8.8 × 10^−2^	1.0
	hsa01250:Biosynthesis of nucleotide sugars	5	1.1	1.2 × 10^−2^	4.8 × 10^−1^
	hsa00620:Pyruvate metabolism	5	1.1	2.6 × 10^−2^	8.0 × 10^−1^
	hsa00650:Butanoate metabolism	4	0.9	2.9 × 10^−2^	8.0 × 10^−1^
	hsa00640:Propanoate metabolism	4	0.9	4.1 × 10^−2^	9.3 × 10^−1^
	hsa00512:Mucin type O-glycan biosynthesis	4	0.9	5.5 × 10^−2^	1.0
	hsa00260:Glycine, serine and threonine metabolism	4	0.9	7.2 × 10^−2^	1.0
	hsa02010:ABC transporters	4	0.9	9.4 × 10^−2^	1.0
	hsa00100:Steroid biosynthesis	3	0.6	8.3 × 10^−2^	1.0
	hsa00900:Terpenoid backbone biosynthesis	3	0.6	9.8 × 10^−2^	1.0

## Data Availability

Not applicable.

## Supplementary Materials

The following supporting information can be downloaded at: https://www.mdpi.com/article/10.3390/ijms23105653/s1.
